# DNA Origami and Its Applications in Synthetic Biology

**DOI:** 10.1002/advs.202513357

**Published:** 2025-11-27

**Authors:** Yaning Fang, Xuexin Chen, Qingsheng Qi, Min Lin, Quanfeng Liang

**Affiliations:** ^1^ State Key Laboratory of Microbial Technology Shandong University Qingdao 266237 P. R. China; ^2^ College of Agriculture Henan University Kaifeng 475001 P. R. China

**Keywords:** cell‐free, DNA origami, gene editing, synthetic biology

## Abstract

In recent years, DNA origami technology has advanced rapidly as a groundbreaking method for nanomanufacturing. This technology takes advantage of the unique base‐pairing characteristics of DNA, and has significant advantages in constructing spatially ordered and programmable nanostructures. This capability aligns with synthetic biology's core principle of mimicking, extending, and reconstructing natural biological processes by modularly assembling artificial systems. This article provides a comprehensive overview of DNA origami's innovative applications across various domains, including cell membrane surfaces, intercellular communication, intelligent biosensing, and precise gene editing, progressing from the extracellular to the intracellular environment. Finally, this review highlights the synergistic interaction between this technology and cell‐free synthetic biology, achieved through the integration of in vitro assembly and cellular regulation, thereby opening new pathways for the rational design of artificial life systems.

## Graphical abstract

This review provides a comprehensive summary of recent advancements in DNA origami for synthetic biology, emphasizing two key areas: the precise construction and dynamic regulation of extracellular‐to‐intracellular, and the synergistic integration with cell‐free systems. By combining in vitro assembly with cellular regulation, DNA origami synergizes with cell‐free platforms, opening new pathways for innovative approaches in artificial life design. The review also addresses the challenges and future prospects of this field.

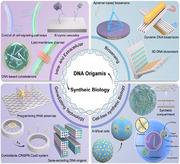

## 1 Introduction

DNA, widely acknowledged as the molecular blueprint of life, has extended beyond biological systems to drive innovative non‐biological applications, facilitated by advances in nucleic acid synthesis and sequencing technologies. The structural versatility, programmable design flexibility, and molecular addressability inherent in DNA architecture offer significant advantages for applications in nanotechnology, biomedical engineering, and synthetic biology.^[^ ^1^, ^2^, ^3^, ^4^ ^]^ Among these innovations, DNA origami technology stands out as a transformative advancement, enabling the precise fabrication of complex nanostructures through sequence‐specific self‐assembly. It has become an indispensable tool for investigating biological mechanisms and analyzing the functions of various biological systems.

Although much of the research on DNA origami technology remains conceptual, its value has been demonstrated across various interdisciplinary fields. It has become a critical supporting technology for synthetic biology due to its unique advantages, including high designability, precise addressability, biocompatibility, and modular assembly.^[^ ^5^ ^]^ Existing literature on DNA nanostructure construction primarily focuses on utilizing Watson‐Crick base pairing and pi‐pi stacking interactions to form double‐helical DNA domains of defined lengths.^[^ ^6^ ^]^ In DNA origami, multiple short DNA strands, typically 15 to 60 nucleotides long, serve as staple strands, while the long single‐stranded scaffold is folded into the target structure via a one‐pot annealing process. DNA molecules exhibit ideal properties for synthetic biology applications. First, DNA is programmable, with Watson‐Crick base pairing ensuring sequence specificity and directional hybridization with high fidelity. The stability of DNA binding is influenced by controllable parameters such as temperature, ionic strength, and DNA concentration. Second, DNA is chemically and biologically stable, exhibiting good biocompatibility. It can penetrate cells and be modified at specific sites. Third, DNA origami enables the creation of artificial functional cells within synthetic cells or on their membranes. Furthermore, more complex DNA origami structures can introduce multifunctionality and structural diversity into synthetic biology. Synthetic biology leverages the three primary characteristics of DNA: complementarity between strands in the helical form, polymerization (and its modifications), and sequence‐specific recognition.^[^ ^7^ ^]^

**Figure 1:**
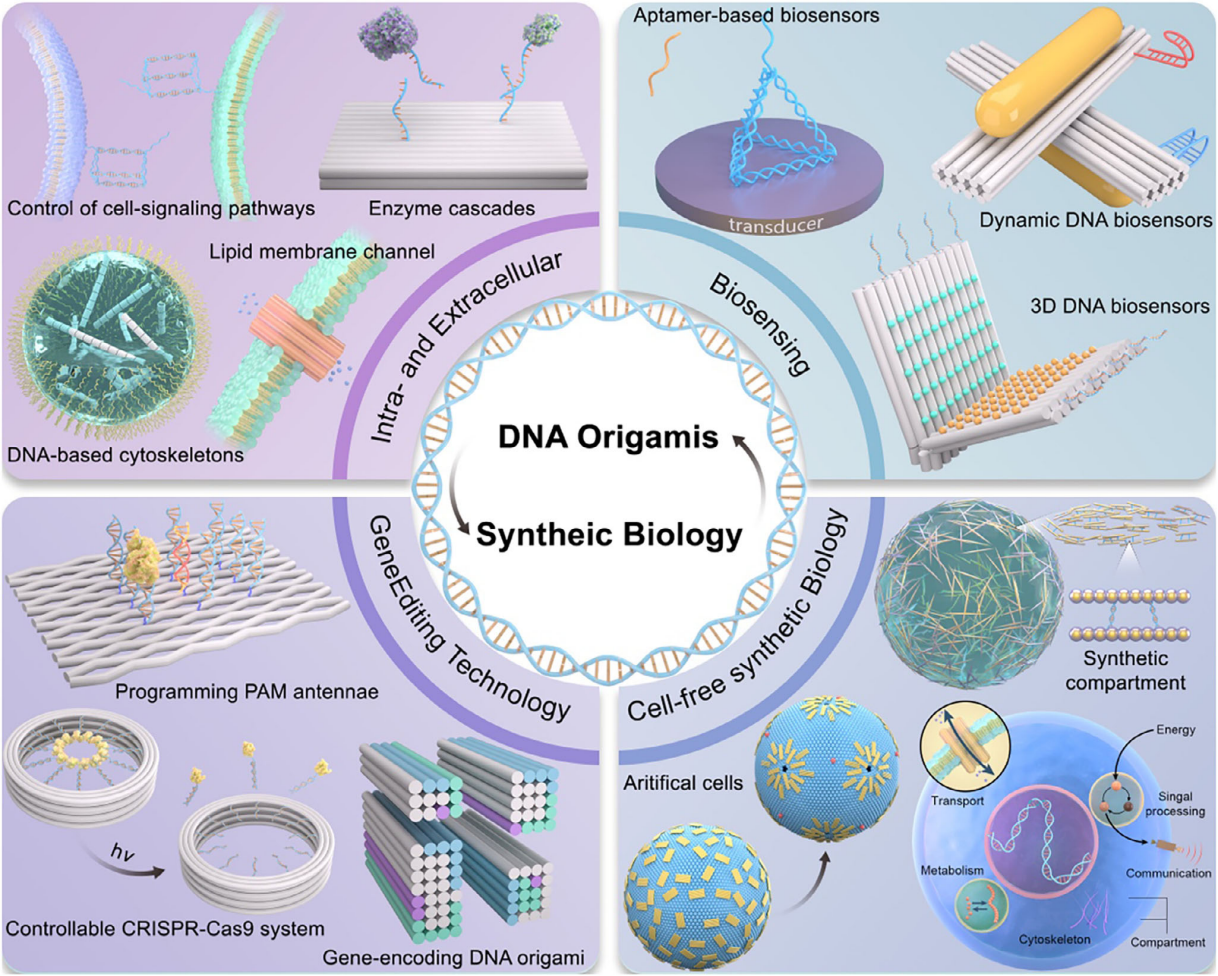
DNA origami and its applications in synthetic biology. Applications of DNA origami in cell membranes, intercellular communication, and cell structure (top left) include control of cell‐signaling, lipid membrane channel and cytoskeletons, etc. DNA origami‐based sensors transition from one‐dimensional to 3Dand even dynamic DNA sensors (top right). Combined application of DNA origami and CRISPR systems, with representative schematic diagrams shown in the figure (bottom left). The integration of DNA origami with cell‐free synthetic biology facilitates the exploration of life's mysteries and the construction of synthetic cells (bottom right).

The core objective of synthetic biology is to engineer and construct living systems, endowing them with novel or naturally inaccessible functions to address major societal challenges in health, environment, energy, and materials. Within this framework, biosensing constitutes a critical information input step, necessitating the transmission of signals across the cell membrane barrier. From extracellular signal transduction and intercellular communication to membrane surface engineering, and further into intracellular structural engineering involving the cytoskeleton, protein networks, and enzyme cascades‐culminating in programmable intracellular processes like gene editing and expression—a complex hierarchy of biological events unfolds from the outside in. DNA origami technology demonstrates significant application potential across these sophisticated processes. Concurrently, the diverse molecular components and mechanisms intrinsic to these processes provide vital foundations for cell‐free synthetic biology research. Therefore, this article explores the application of DNA origami in synthetic biology along two key dimensions: from extracellular to intracellular systems, and within cell‐free synthetic biology platforms (**Figure** **1**). The development of DNA origami structures is briefly reviewed, followed by an in‐depth discussion of the integration of DNA origami with cell membranes, intracellular structures, intercellular communication, biosensors, and gene editing systems. Finally, current technical challenges, such as in vivo stability and large‐scale preparation, are analyzed, along with future development directions. This review aims to provide theoretical support for the deeper integration of DNA origami technology with synthetic biology and to foster innovative applications in fields such as biomanufacturing and metabolic engineering.

## 2 DNA Origami Structure

Due to Watson‐Crick base pairing, DNA strands exhibit high sequence‐dependence in hybridization, making them ideal molecular building blocks for synthesizing nanostructures with strong programmability and modular functionality. Various strategies and consistent assembly techniques have been developed to control DNA nanostructures, achieving increasingly complex designs. Among these, DNA origami technology stands out as a promising approach in DNA nanotechnology. First proposed by Rothemund in 2006, DNA origami laid the foundation for subsequent research into complex DNA nanostructures.^[^ ^8^ ^]^ By using viral genomes such as M13mp18 (≈7249 nt) as scaffolds, DNA origami involves folding these long strands with more than 200 short oligonucleotides, known as “staple strands,” to form classic 2D shapes such as squares, smiling faces, and five‐pointed stars. 2D DNA origami has since found numerous applications.

Due to their greater complexity and programmability, 3D DNA origami structures are capable of utilizing crystals as scaffolds for organizing proteins and other biomolecules, thus addressing challenges in macromolecule crystallization.^[^ ^9^ ^]^ Several methods have been developed to expand DNA origami into 3D structures. Three main strategies are employed: the first involves connecting planar 2D DNA origami to form 3D structures. For instance, Yan et al. successfully constructed a hollow tetrahedral structure by folding four connected triangular planes.^[^ ^10^ ^]^ Komiyama and colleagues used a similar strategy to achieve the preparation of DNA origami cube structures for the first time. With the help of the added DNA strands, the “lid” of this 3D structure can be selectively opened or closed.^[^ ^11^ ^]^ This enables the encapsulation of molecules such as proteins, enzymes, and metal nanoparticles, broadening the potential applications of DNA origami. The second strategy constructs 3D DNA origami structures with curved surfaces by introducing cross structures and guiding surface formations.^[^ ^12^ ^]^ The third method involves creating wireframe 3D DNA origami by transforming the structure into a triangular 3D grid, using a routing algorithm to trace the structural outline with DNA origami scaffolding.^[^ ^13^ ^]^

Despite substantial progress in structural DNA origami, the deterministic assembly of mesoscale DNA origami architectures, ranging from micrometers to millimeters, remains a critical challenge that limits their application in synthetic biological systems, particularly in protocell engineering and synthetic cellular chassis development. Current limitations are primarily attributed to scaffold length constraints and imperfect hierarchical assembly control. However, several innovative approaches have recently emerged to address these challenges: (I) Modular tile scaling, exemplified by Wang et al.’s orthogonal chemical linkage strategy,^[^ ^14^ ^]^ introduces a branching staple structure into the conventional M13 scaffold system, enabling one‐pot synthesis of programmable super‐DNA origami with controllable tile multiplicity. This method achieves higher yields (>80%) compared to the conventional two‐step co‐assembly system, which yields less than 10%. Yao et al. proposed a strategy using submicrometer‐scale (meta‐DNA) building blocks at the micrometer scale to form diverse and complex structures, such as meta‐dobby junctions, 3D polyhedrons, and various 2D/3D lattices.^[^ ^15^ ^]^ (II) A novel scaffold engineering technique enables the fabrication of sequence‐tailored scaffolds with adjustable length and programmable curvature, using de novo design to create scaffolds with specific local and global sequence features that resist strain.^[^ ^16^ ^]^ (III) Modular addressable assembly: Weck et al. marked an important leap from “customized assembly” to “universal fabrication” by designing a modular DNA origami (moDON) with tens of thousands of different connection site configurations, and a large number of orthogonal connections that allow for one‐step assembly of a variety of superstructures, which will provide an opportunity to realize programmable macroscopic life materials for synthetic biology.^[^ ^5^ ^]^ These multiscale structural programming strategies have established new design principles for DNA‐based metamaterials, addressing key challenges related to assembly yield and structural integrity. They are progressively being applied in synthetic biology, nanophotonics, and biomedicine. Future breakthroughs may rely on the integration of cross‐disciplinary technologies, such as CRISPR‐edit‐assisted assembly, with automated experimental platforms.

## 3 DNA Origami Technology and Synthetic Biology

The rational design of microorganisms has long been an ambitious goal. With the rise of genomics and systems biology in the 1990s, synthetic biology emerged as a field of study.^[^ ^17^ ^]^ Synthetic biology adopts a bottom‐up approach to construct biological components and systems to mimic or even in some scenarios replace natural living systems. The precise controllability at the nanoscale enables DNA origami to be used for the creation of “standardized” and “modular” tools, laying the foundation for future engineering platforms built on these tools. DNA offers several advantages over other polymers: First, DNA molecules interact with each other primarily according to the Watson‐Crick base pair rule, which allows for predictable and programmable construction of high‐fidelity DNA materials. Second, DNA oligonucleotides can be chemically synthesized using standard phosphoramidite chemistry with high purity and low cost, and can be further modified with functional groups internally or terminally. Third, DNA can be tailored to exhibit the desired rigidity or flexibility by choosing single‐stranded DNA (ssDNA), double‐stranded DNA, or combinations of both. Fourth, DNA molecules (i.e., DNA aptamers) can in principle be selected from synthetic libraries with high binding specificity and affinity against any molecule, from large proteins to small chemicals. DNA also offers additional benefits such as reversibility, stability, biocompatibility, and biodegradability. These properties position DNA origami as a promising candidate for applications in synthetic biology (**Figure** **1**). Extracellularly, DNA nanostructures anchored to the cell membranes enable spatiotemporally precise, real‐time monitoring of microenvironmental dynamics. At the membrane interface, DNA origami‐lipid hybrid systems replicate the functional properties of natural membrane proteins and transmembrane complexes. Intracellularly, DNA origami scaffolds facilitate the precise engineering of synthetic protein networks, creating high‐fidelity, predictably addressable platforms to reconstitute metabolic pathways. DNA origami‐based biosensors exhibit expanded target specificity, enhanced durability, and reduced production costs compared to conventional systems. Moreover, their synergistic integration with CRISPR systems permits programmable spatial organization of protospacer adjacent motif (PAM) microenvironments, facilitating mechanistic studies of DNA‐editing processes. Notably, in vivo structural reconfiguration of DNA origami devices can directly modulate gene expression.

### 3.1 Surface Engineering of Cell Membranes

The structure and function of cell membranes are critical for cellular survival and physiological activities, regulating essential processes such as selective permeability, molecular recognition, signal transduction, intercellular adhesion, and motility. Surface engineering of cell membranes offers new tools to modulate cell‐environment communication, redefine cellular functions, and better understand various cellular processes.^[^ ^19^ ^]^ A particularly attractive strategy lies in engineering cellular behavior or their microenvironmental interactions through precise monitoring or synthetic control of membrane dynamics. DNA origami‐based nanostructures have emerged as a versatile platform for cell surface engineering (**Table** **1**). By utilizing site‐specific chemical conjugation techniques, functional moieties such as oligonucleotide tags or lipid molecules can be hybridized to predefined sites on DNA origami scaffolds, enabling programmable interactions with lipid bilayers.

**Table 1:** Summary of applications and challenges of multifunctional DNA origami in extracellular‐intracellular coordination.

	Applications	Advantages	Problems	Refs.
Surface engineering of cell membranes	Guiding membrane assembly	DNA origami have richer structural diversity. DNA origami enables precise positioning of leading hydrophobic groups (LHGs) at specific sites for controlled structural assembly. Limitations of the DNA origami framework lead to more homogeneous synthesized liposomes.	DNA origami's environmental sensitivity constrains real‐world implementation. Immature DNA origami scale‐up production techniques have hindered industrial‐scale applications. Currently not genetically encodable.	[22]
	Constructing DNA membrane channels	DNA membrane channels of any shape can be designed by simple de novo. Easier to fold than engineered proteins. Controlled at nanometer precision, breaking the size limit of artificial membrane channel construction.		[23, 24]
	Regulating lipid membranes	Achieving molecular recognition‐initiated DNA assembly to mimic the dynamic behavior of membrane proteins, thereby enabling manipulation of cellular interactions in response to environmental changes.		[27, 28]
Intercellular communication engineering	Extracellular microenvironment monitoring	DNA origami's affinity for target analytes allows for structural transformations, facilitating the detection of minute amounts of substances secreted into the extracellular environment, thereby providing a more accurate understanding of the cell's state.		[32]
Intercellular communication engineering	Mimicking the natural signaling pathway	Characterizing DNA origami structures allows for precise control of signal transduction systems, thereby enabling the management of higher‐order complexity in cell mimicry. This makes it possible to artificially regulate physiological and pathophysiological processes, addressing significant scientific challenges.	DNA origami relies on specific ion concentrations (e.g., Mg^2+^) to maintain structural stability, and the complex environment in cells may face degradation problems. Existing microscopy techniques struggle to track 3Dnanoscale dynamic processes in real‐time, limiting the ability to optimize systems effectively.	[37]
Intracellular structure engineering	DNA origami builds the cytoskeleton	DNA origami enables the construction of programmable, reversible, versatile, and functional cytoskeletons with atomic‐level precision, serving as synthetic cell models.	The complex ionic environment within the cell, along with the presence of nucleases, makes DNA prone to degradation, compromising the stability of the cytoskeleton. Minute delays in DNA strand displacement lead to temporal mismatches in physiological dynamics, requiring acceleration of the response through the development of fast switching mechanisms. High doses of nucleic acid nanostructures may induce potentially immunogenic stimuli.	[41]
	DNA origami builds protein networks, controls enzyme cascade reactions	DNA origami scaffolds enable the creation of programmable protein networks with high precision, predictability, and addressability. The distance of intermediate proteins and the spatial position of the enzyme are controlled with nanoscale precision to regulate the reaction between the enzyme and the substrate.		[45, 49]

**Figure 2:**
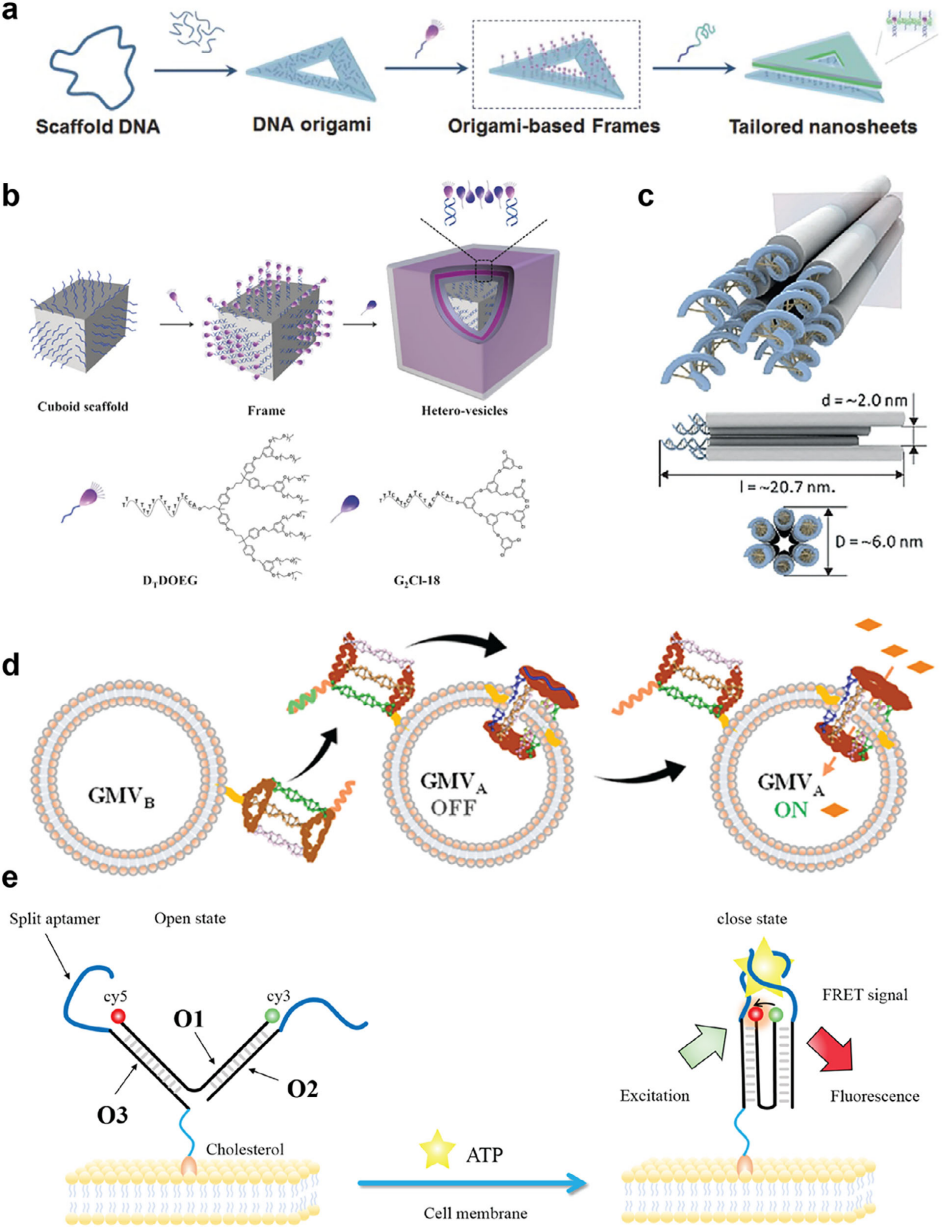
a) A guiding mechanism for fabricating monodispersed and shape‐defined nanosheets based on a framework assembly strategy using DNA origami as the scaffold. Reproduced with permission.^[^ ^20^ ^]^ Copyright 2016, WILEY‐VCH Verlag GmbH & Co. KGaA. b) PolyT‐ amphiphilic dendritic molecules (DTDOEG) hybridize with poly(A) extension sequences on cuboid origami, guiding the main amphiphilic molecule (G2Cl‐18) to assemble through hydrophobic interactions, ultimately completing the formation of heterogenous vesicles around the framework. Reproduced with permission.^[^ ^21^ ^]^ Copyright 2017, Wiley‐VCH Verlag GmbH & Co. KGaA. c) Conformation of the designed 6HB. Adapted with permission.^[^ ^23^ ^]^ Copyright 2018, Wiley‐VCH Verlag GmbH & Co. KGaA. d) Design and construction of a bionic Artificial signal transmission system. GMV_A_ acts as a receptor and an actuator, respectively modified by DNA triangular prism and DNA nanopore (embedded in the cell membrane), while GMV_B_ acts as a stimulator, modified by DNA triangular prism. Among them, the short blue strand on the upper plane of the DNA nanopore is a lock strand, which closes the entrance of the nanopore. When it undergoes a fulcrum strand shift reaction with the foreign complementary strand and is released from the nanopore, it will reopen the channel. Adapted with permission.^[^ ^36^ ^]^ Copyright 2020, American Chemical Society. e) Cholesterol‐anchored DNA nanoswitches with dimeric sensing units for real‐time extracellular ATP monitoring. Reproduced with permission from.^[^ ^35^ ^]^ Copyright 2019, American Chemical Society.

Zhou et al. demonstrated the 2D assembly of amphiphilic DDOEG molecules through a framework‐guided assembly (FGA) strategy, using DNA origami as programmable templates (**Figure** **2**a).^[^ ^20^ ^]^ The DDOEG molecules, anchored to the origami through DNA hybridization, serve as leading hydrophobic groups (LHGs), forming a 2D framework structure above the DNA origami. The local concentration of hydrophobic groups within this framework is significantly higher than in the surrounding solution. Hydrophobic‐hydrophobic interactions drive the absorption of additional DDOEG or other amphiphilic molecules into this domain, reducing the total free energy and progressively forming homogeneous or heterogeneous nanosheets above the DNA origami. Notably, the authors established a template‐directed assembly mechanism, where the pre‐patterned LHG distribution on DNA origami dictates the morphology of the resulting nanostructures. Expanding on this, the FGA strategy was applied to engineer 3D DNA origami frameworks, successfully directing the assembly of heterogeneous vesicles with controlled compositions (Figure 2b).^[^ ^21^ ^]^ These breakthroughs not only achieve biomimetic reconstruction of lipid membrane morphologies using high‐precision DNA origami but also provide mechanistic insights into how natural proteins regulate membrane dynamics to coordinate cellular processes.

Membrane channels are vital for mediating cellular exchange with the extracellular environment, maintaining homeostasis through selective molecular transport essential for metabolic regulation. Recent advancements in DNA origami‐lipid membrane hybrid systems have enabled the creation of sophisticated nanostructures that emulate membrane‐anchored proteins and transmembrane complexes. Given the size‐dependent gating mechanisms inherent to natural channels, DNA origami offers exceptional potential for biomimetic channel engineering by precisely controlling nanopore dimensions. These membrane‐bound DNA architectures modulate lipid bilayer morphology, mechanical properties, and biological functionality through two distinct mechanisms: (I) dynamic membrane remodeling and (II) intrinsic structural programmability. Current DNA‐based membrane channels are classified into two categories: static nanochannels and stimuli‐responsive dynamic systems. Langecker et al. pioneered the first fully synthetic transmembrane channel using scaffolded DNA origami, integrating it into lipid bilayers through cholesterol‐mediated anchoring.^[^ ^22^ ^]^ This breakthrough set the foundation for engineering ion flux‐driven nanodevices that mimic natural membrane machinery, such as ion pumps and transport proteins. Meanwhile, dynamic DNA nanochannels, exemplified by Liu et al.’s electrostatically tunable hexagonal bundle (6HB) nanotubes, demonstrate environmental adaptability by modulating molecular transport through real‐time electrostatic repulsion forces (Figure 2c).^[^ ^23^ ^]^

Cholesterol‐functionalized DNA origami further enhances programmable membrane interactions, supporting stable anchoring and lateral diffusion across lipid bilayers.^[^ ^24^ ^]^ The ability of DNA nanostructures to induce controlled vesicle deformation represents a significant advancement in artificial cellular component engineering. Although current assembly processes are largely irreversible, dynamic reconfiguration can be achieved through strand displacement reactions or stimuli‐responsive DNA systems activated by ionic gradients, pH changes, or light inputs. Additionally, hierarchical assembly of DNA origami enables the creation of multi‐channel architectures with larger nanopores, facilitating systematic studies of transmembrane transport phenomena.

In synthetic biology, designing artificial cells capable of sensing and processing signals from the cellular microenvironment is essential. Cellular microenvironments fundamentally regulate physiological processes through spatiotemporal control of metabolic activities and intercellular signaling.^[^ ^25^ ^]^ DNA origami technology can construct biological circuits in different scenarios based on its advantages. A seminal demonstration by Liu et al. engineered an artificial signaling feedback network on giant membrane vesicle (GMV) protoplasts.^[^ ^26^ ^]^ By iteratively activating and deactivating DNA strand displacement circuits, this system achieved cyclic resetting of membrane states, providing a robust platform to study dynamic signal transduction mechanisms. Moreover, ATP‐responsive DNA probes anchored via cholesterol tags enable molecular recognition‐driven assembly processes that biomimetically replicate membrane protein dynamics.^[^ ^27^ ^]^ Such DNA‐mediated systems allow precise manipulation of cell‐matrix interactions in response to microenvironmental changes. Integrating DNA origami nanostructures into lipid bilayers positions these constructs as key elements in synthetic cellular systems. Emerging paradigms extend beyond individual artificial cells to multicellular architectures, where spatially organized vesicle networks could coordinate material exchange and chemical signaling through DNA‐programmed communication protocols. This capability suggests broader applications in membrane‐mediated processes, including collective metabolite transport and synchronized enzymatic cascades.

In conclusion, the advantages of DNA origami, such as programmability, addressability, and good biocompatibility, provide a powerful tool for precise nanomanipulation on the cell membrane surface, a key interface of life activities. Particularly, DNA origami structures exhibit extensive application potential in guiding the precise and controllable synthesis of artificial membrane structures, constructing transmembrane channels, sensing and detecting dynamic membrane surface events, and regulating intercellular interactions. Addressing the challenges associated with DNA origami will focus on artificial intelligence (AI) assisted optimization of structural design, cost reduction for scalable production, and mitigation of immunogenicity. Future advancement will concentrate on overcoming these existing hurdles and expanding the boundaries of innovation. This progress aims to transform the cell membrane from a passive carrier interface into a programmable, intelligent platform with active functionalities, ultimately achieving a DNA origami – cell membrane system capable of integrated environmental perception, robust logical operations, and response output. The future development of DNA origami applications on cell membranes hinges critically on deep interdisciplinary integration spanning materials science, chemical biology, biophysics, bioengineering, computational simulation, and medicine. Leveraging DNA origami for cell surface engineering demonstrates significant promise in diverse fields such as tissue engineering, cancer immunotherapy, biosensing, cell regulation, and targeted cell delivery.

### 3.2 Intercellular Communication Engineering

Cell communication with the extracellular environment is crucial for various life processes, and various life activities depend on communication with the external environment, such as metabolism, proliferation and apoptosis of the cells themselves.^[^ ^28^, ^29^ ^]^ DNA's intrinsic characteristics—such as dynamic responsiveness to external stimuli, structural reconfigurability, and programmable hybridization—make it a versatile modular platform for molecular recognition and adaptive nanostructure engineering (Table 1).

Cells' inherent sensitivity to their microenvironment makes them vulnerable to pathological alterations under abnormal extracellular conditions.^[^ ^30^ ^]^ DNA nanodevices anchored to plasma membranes offer distinct advantages over traditional culture medium analysis, enabling in situ microenvironment monitoring with spatiotemporal precision.^[^ ^31^ ^]^ Yang et al. developed a ratiometric pH‐sensing system using dual fluorescent amphiphilic lipid‐DNA probes: a pH‐responsive fluorophore paired with a pH‐insensitive reference dye, allowing reversible detection within physiological pH ranges (6.0–8.0).^[^ ^32^ ^]^ Expanding on this approach, Zeng et al. introduced cholesterol‐tethered DNA tweezers incorporating i‐motif structural switches for dynamic pH monitoring (5.0–7.5) through reversible conformational transitions.^[^ ^33^ ^]^ Additionally, DNA‐based nanoprobes are gaining popularity for detecting metal ions, which play a pivotal role in cell signaling and communication, leveraging DNA sequences' ability to recognize metals.^[^ ^34^ ^]^ Bioactive molecules, vital for cellular communication with the extracellular environment, have also been explored. Yuan et al. demonstrated real‐time extracellular ATP tracking using cholesterol‐anchored DNA nanoswitches with dimeric sensing units (Figure 2e).^[^ ^35^ ^]^

Beyond single‐cell monitoring, DNA nanotechnology enables the programmable manipulation of multicellular communication networks. Cell membrane‐anchored immobilized DNA platforms are highly programmable and flexible, showing great promise in mimicking and manipulating cellular interactions. A seminal study by Yang et al. demonstrated this potential through the design of an artificial DNA‐based signaling system that mimics intercellular communication dynamics (Figure 2d).^[^ ^36^ ^]^ The system relies on a DNA toehold‐mediated strand displacement reaction, which opens a DNA membrane channel to release information, enabling communication between two communities. Synthetic transmembrane channels are activated to allow ion influx. By emulating natural signaling pathways, this DNA origami‐based model provides a valuable tool for studying intercellular communication and offers a modular framework for constructing physiological processes that mimic natural biological systems. The integration of dynamic DNA origami nanostructures with synthetic biology principles opens new avenues for decoding spatial‐temporal patterns in multicellular signaling, and developing programmable therapeutics targeting pathological communication networks.

DNA origami offers unique advantages for intercellular communication engineering, including sensitive real‐time environmental monitoring, precise control of signal transmission, and amplification of biological signals. Despite facing challenges such as in vivo stability, delivery efficiency, and large‐scale production, the field is rapidly advancing toward enhanced stability, smarter functionalities, improved delivery efficiency, and greater clinical translatability. Design a cascaded communication network involving multiple cell types, simulate the tissue microenvironment, and construct a more intelligent and complex inter‐cell communication network based on DNA origami. These advances are also critically relevant to metabolic engineering, where dynamic regulation triggered by external signals enables the redirection of metabolic flux toward specific pathways based on cellular inputs. This strategy holds potential for increasing the titer of valuable chemicals in microbial production strains.^[^ ^83^ ^]^ Looking forward, DNA origami stands as a powerful platform for intercellular communication engineering, promising to simulate natural signal transduction pathways and drive transformative breakthroughs in diverse fields such as tissue engineering, metabolic engineering, synthetic biology, and precision medicine.

### 3.3 Intracellular Structure Engineering

The intricate organization of living cells fundamentally differs from simplified cell‐free molecular systems, with the cytoskeleton serving as the central architectural framework that governs cellular morphogenesis, mechanical adaptation, and stimulus‐responsive behaviors.^[^ ^37^ ^]^ This dynamic protein network enables spatial compartmentalization, which is essential for executing complex biological processes, making cytoskeletal engineering a fundamental component in the development of bottom‐up synthetic cells (Table 1).

**Figure 3:**
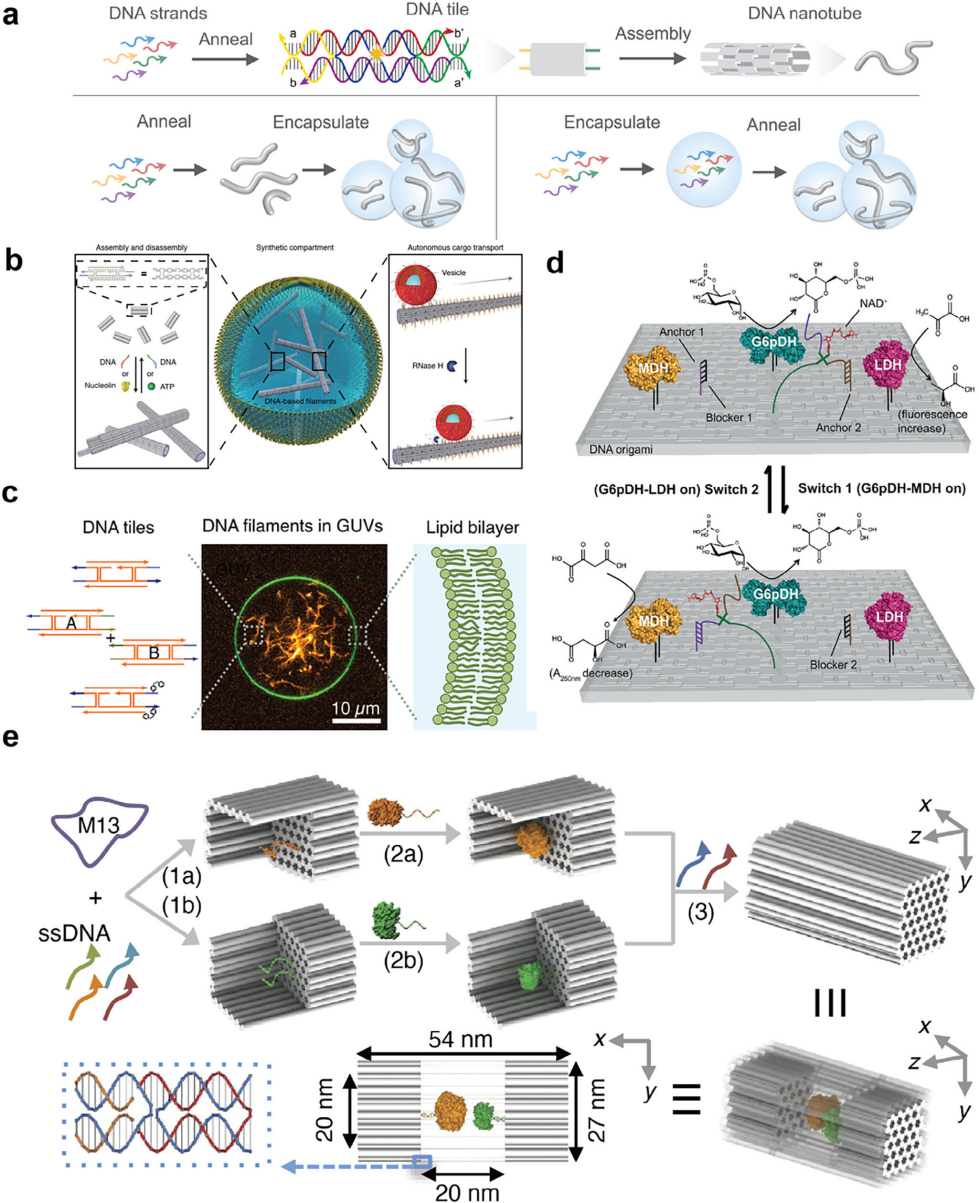
a) Schematic of DNA tiles and nanotubes. Adapted under terms of the CC‐BY license.^[^ ^41^ ^]^ Copyright Year 2021, The Author(s), published by Springer Nature. b) Functional DNA‐based cytoskeletons for synthetic cells. Reproduced under terms of the CC‐BY license.^[^ ^40^ ^]^ Copyright Year 2022, The Author(s), published by Springer Nature. c) Schematic of a GUV containing a DNA cytoskeleton comprised of filaments. The DNA cytoskeleton can be reversibly assembled within the GUV to form a synthetic cell model encapsulated by a lipid bilayer. Reproduced under terms of the CC‐BY license.^[^ ^42^ ^]^ Copyright Year 2022, The Author(s), published by American Chemical Society. d) A DNA origami system regulates two enzyme pathways directionally through the control of NAD^+^ substrate channeling by specifically shifting NAD^+^ between the two enzyme pairs (G6pDH–MDH and G6pDH–LDH). Reproduced with permission from.^[^ ^53^ ^]^ Copyright 2016, WILEY‐VCH Verlag GmbH & Co. KGaA. e) Schematic diagram of the structural design of DNA nanocages encapsulating enzymes and their encapsulation of a pair of GOx (orange) and HRP (green) enzymes. Adapted under terms of the CC‐BY license.^[^ ^51^ ^]^ Copyright Year 2016, The Author(s), published by Springer Nature.

DNA origami nanotechnology stands out as one of the most promising approaches for constructing cytoskeletons, owing to the inherent sequence specificity, addressability, and programmability of DNA.^[^ ^38^, ^39^ ^]^ Pioneering work by Kurokawa et al. demonstrated cytoskeletal mimicry through Y‐motif DNA nanostructures self‐assembled via electrostatic interactions with cationic lipid membranes, forming gel‐like networks through sticky‐end annealing.^[^ ^38^ ^]^ Subsequent advancements have tackled the complexity of the cytoskeleton through hierarchical assembly strategies: Zhang, for instance, constructed a tubular assembly system using DNA origami monolithic prototypes.^[^ ^39^ ^]^ By designing molecular‐level structures, DNA origami objects based on 32HB (helical bundles) can form micrometer‐long tubular structures through complementary side‐patterned splicing and head‐to‐tail blunt‐end stacking. The inherent versatility of the cytoskeleton presents significant challenges in creating bottom‐up synthetic cells. Zhan et al. extended this paradigm through tile‐based assembly of hollow DNA nanotubes with tunable mechanical properties (**Figure** **3**b).^[^ ^40^ ^]^ Siddharth Agarwal et al. pioneered studies on DNA nanotube assembly in confined environments, where DNA nanotubes are dynamically assembled and disassembled when preformed DNA tiles are encapsulated in droplets of water‐in‐oil emulsion (Figure 3a).^[^ ^41^ ^]^ Kevin Jahnke et al. demonstrated the creation of a programmable, reversible, multifunctional cytoskeleton made from DNA within giant single‐membrane lipid vesicles (GUVs) as a synthetic cell model (Figure 3c).^[^ ^42^ ^]^

Beyond structural mimicry, DNA origami scaffolds enable the precision engineering of artificial protein networks, which is essential for the spatiotemporal control required in metabolic systems. The DNA origami structure offers a large surface area, and by modifying the backbone to attach functional groups at specific sites, it allows the construction of highly precise, predictable, and addressable protein network platforms. The key to constructing DNA origami protein networks lies in the binding of proteins to DNA. Various methods have been developed for protein binding to DNA origami, including antigen‐antibody interactions, aptamer‐protein binding, and small molecule cross‐linkers. These methods are primarily classified into noncovalent and covalent conjugation strategies.^[^ ^43^ ^]^ DNA origami tiles have been used for the precise localization of proteins based on their attachment to specific positions on the DNA scaffold.^[^ ^44^ ^]^ Saccà et al. demonstrated selective multi‐protein patterning on 2D DNA‐origami through position‐specific biotin‐streptavidin and digoxigenin‐antibody interactions.^[^ ^45^ ^]^ Additionally, an approach centered on protein composition can accelerate the specific assembly of proteins on DNA nanostructures. Nakata et al. advanced this technique by using zinc finger protein‐mediated site‐specific anchoring of engineered enzymes, establishing a modular framework for synthetic metabolon construction.^[^ ^46^ ^]^

Enzyme cascade engineering exemplifies the functional potential of DNA‐origami scaffolds (Figure 3d). In enzyme cascade reactions, DNA origami scaffolds regulate protein‐substrate interactions by precisely controlling the distance and spatial positioning of intermediate proteins, thereby forming artificial cascades. Leveraging the nanoprecision and spatial addressability of DNA origami, protein networks have been utilized to construct and regulate enzymatic cascades, including glucose oxidase and horseradish peroxidase (GOx‐HRP),^[^ ^47^, ^48^ ^]^ G6pDH‐LDH,^[^ ^49^ ^]^ the xylose metabolic pathway,^[^ ^50^ ^]^ and caspase‐9 dimerization.^[^ ^53^ ^]^ Ngo et al. systematically investigated xylose‐to‐xylulose‐5‐phosphate conversion efficiency as a function of inter‐enzyme distances in three‐enzyme DNA scaffolds.^[^ ^50^ ^]^ Fu et al. organized discrete glucose oxidase (GOx) and horseradish peroxidase (HRP) enzyme pairs on specific DNA origami tiles, controlling inter‐enzyme spacing and positioning, while restricting the diffusion of intermediates to the 2D surface of the ligase, thereby enhancing enzyme cascade activity.^[^ ^48^ ^]^ Linko et al. expanded this concept into a 3D tubular reactor, designing a spatially addressable tubular DNA origami nanoscale reactor that efficiently facilitated the GOx/HRP enzyme cascade reaction within the tube.^[^ ^52^ ^]^ Zhao et al. utilized DNA nanocages to encapsulate metabolic enzymes, enhancing their activity and protecting them from protease‐mediated degradation (Figure 3e).^[^ ^51^ ^]^ Furthermore, many natural enzyme cascades depend on substrate channels. Instead of altering enzyme distances, enzyme reactions can also be modulated by adjusting the position of the substrate channel in the cascade. For instance, Yan et al. pioneered substrate‐channeling strategies, employing NAD^+^‐modified ssDNA tethers to regulate competing metabolic pathways (G6pDH‐MDH and G6pDH‐LDH) via controlled cofactor shuttling (Figure 3d).^[^ ^53^ ^]^ These advances represent a paradigm shift in metabolic engineering: DNA origami platforms allow for the rational manipulation of enzyme spatial relationships and substrate diffusion pathways independent of genetic modifications. By strategically conjugating rate‐limiting enzymes from unmodifiable pathways onto programmable scaffolds, precise control over product flux may be achieved through inter‐enzyme distance optimization, substrate‐channel engineering, and the design of compartmentalized reaction microenvironments.

Although the use of diverse DNA origami structures to organize protein networks and control enzyme cascade reactions has demonstrated capabilities for mimicking natural life systems, deeper theoretical modeling and systematic experimentation are required to fully elucidate the critical parameters influencing DNA origami assembly and function. While its application in intracellular structural engineering remains nascent, DNA origami technology provides a highly programmable platform to execute such tasks with precise control. Looking ahead, leveraging biomimetic designs and the integration of smart materials will enable a transition from static structures to dynamic systems, and from single functions to complex networks. This convergence of DNA origami technology and biology promises the design of synthetic cells, with potential applications spanning biomedicine, synthetic biology, and beyond.

### 3.4 Biosensing

In recent years, DNA origami biosensors have emerged as transformative platforms in analytical biotechnology, utilizing programmable molecular architectures for high‐precision analyte detection. These systems leverage the structural reconfigurability induced by target binding, enabling precise detection of trace substances through mechanochemical signaling.

**Figure 4:**
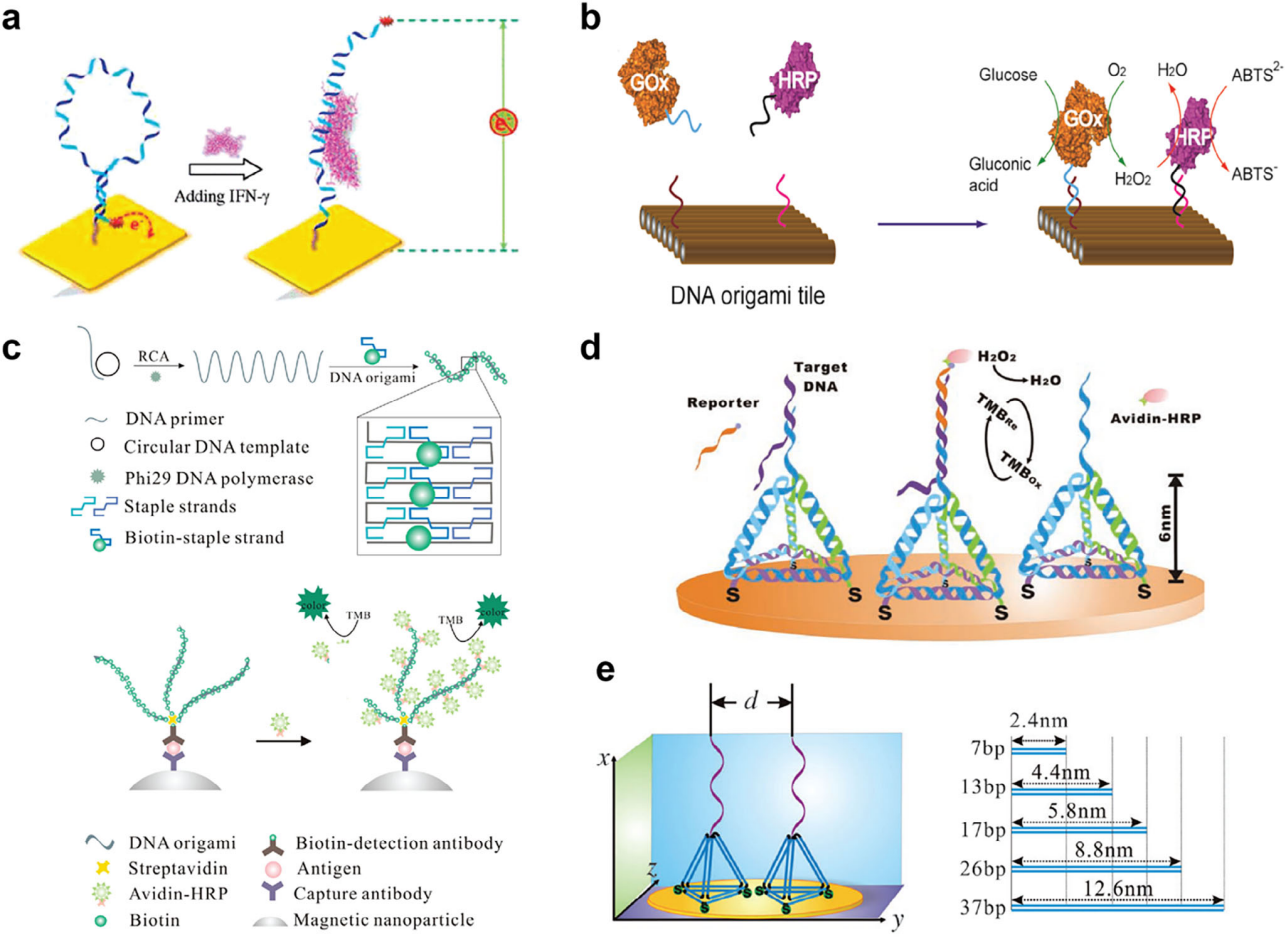
a) Schematic of aptamer‐based electrochemical sensor for IFN‐γ. Reproduced with permission.^[^ ^55^ ^]^ Copyright 2010, American Chemical Society. b) Schematic diagram of discrete GOx/HRP enzymes arranged at controlled intervals and positions on a specific DNA origami structure. Adapted with permission from.^[^ ^48^ ^]^ Copyright 2012, American Chemical Society. c) Schematic diagram of a DNA origami sensor based on rolling circle amplification (RCA) signal amplification. Reproduced with permission.^[^ ^59^ ^]^ Copyright 2014, American Chemical Society. d) Sensor platform based on 3D DNA nanostructures. Reproduced with permission from.^[^ ^60^ ^]^ Copyright 2010, WILEY‐VCH Verlag GmbH & Co. KGaA. e) Precise programmable soft lithography method based on 3D DNA tetrahedral nanostructures (TDNs). Adapted with permission from.^[^ ^61^ ^]^ Copyright 2015, WILEY‐VCH Verlag GmbH & Co. KGaA.

In DNA hybridization biosensors, one‐dimensional (1D) DNA origami can serve as a probe for DNA recognition signals. Single‐stranded oligonucleotide aptamers can bind with high affinity and specificity to a variety of target molecules, including ions (e.g., K^+^, Hg^2+^, and Pb^2+^), small organic molecules (e.g., ATP, amino acids, cocaine, vitamins, and antibiotics), organic dyes, peptides, proteins (e.g., thrombin, and growth factors), and even whole cells or microorganisms (e.g., bacteria).^[^ ^54^ ^]^ Liu et al. have developed an electrochemical DNA aptamer‐based biosensor to detect interferon (IFN)‐γ. The DNA hairpin structure containing the IFN‐γ‐binding aptamer was thiolated, coupled with methylene blue (MB) redox tag, and immobilized on a gold electrode via self‐assembly (**Figure** **4**a).^[^ ^55^ ^]^ Two complementary ssDNAs formed a DNA “stem‐loop” structure as a probe, sensing environmental changes through conformational alterations. For instance, Fan et al. first demonstrated DNA biosensors by detecting electrochemical properties linked to DNA conformational changes (E‐DNA).^[^ ^56^ ^]^

Building on 1D DNA nanostructures, 2D and even 3D DNA origami have demonstrated remarkable advantages in biosensor applications (Table 1). Due to the programmability and addressability of DNA origami, it serves as an ideal platform for biosensors. In 2008, Yan et al. “mounted” long ssDNA on DNA origami as a hybridization probe for molecules such as mRNA in solution and detected it by atomic force microscopy (AFM).^[^ ^57^ ^]^ The same year, the group explored the positional effect of the hybridization reaction, showing that placing the probe at the edge of the DNA origami increased hybridization efficiency for oligonucleotide targets.^[^ ^58^ ^]^ Additionally, the technique has been expanded to include enzyme‐based detection. Wilner et al. pioneered the organization of GOx and HRP enzyme pairs on specific DNA nanostructures with controlled spacing.^[^ ^79^ ^]^ Fu et al. extended GOx and HRP enzyme pairs on specific DNA origami with controlled spacing and positioning, showing that closer proximity enhances enzyme cascade activity (Figure 4b).^[^ ^48^ ^]^ Yan et al. used ssDNA from rolling circle amplification (RCA) as a scaffold strand for DNA origami, which hybridized with three main DNA strands. The DNA strand was conjugated with various enzymes for signal amplification and applied to prostate‐specific antigen detection using magnetic bead ELISA (Figure 4c).^[^ ^59^ ^]^ While DNA origami‐based sensors offer numerous advantages over conventional sensors, 3D DNA origami nanostructures with more complex spatial arrangements stand out in biosensor applications. Pei et al. developed a 3D DNA nanostructure‐based sensor platform for DNA detection, significantly enhancing the spatial localization and accessibility of surface detectors compared to previous DNA sensing architectures (Figure 4d).^[^ ^60^ ^]^ Lin et al. introduced “soft lithography” to modify electrochemical sensors with tetrahedral DNA nanostructures (TDN). By adjusting the size of the TDN, hybridization time was reduced, and efficiency improved. More notably, the detection limit for DNA was adjusted by four orders of magnitude using differential nanostructured electrodes, while polymerase amplification enabled fine‐tuning of sensitivity (Figure 4e).^[^ ^61^ ^]^ The DNA origami biosensor, when integrated with a DNA walker, enables ultra‐sensitive detection. For example, Wang et al. employed enzyme‐driven translocation of a DNA walker along a DNA origami track to achieve linear amplification of fluorescent signals, thereby converting the binding of a single target DNA molecule into the release of multiple fluorescent reporters. Moreover, the walking speed can sensitively discriminate single‐nucleotide mismatches.^[^ ^84^ ^]^ This highly sensitive signal amplification strategy holds significant potential for early cancer diagnosis and biological therapeutics. Additionally, DNA hydrogels, a unique structural form of DNA nanostructures, offer key advantages such as stability, biocompatibility, biodegradability, and tunable versatility, making them ideal for innovative gel‐based applications. Zhang et al. developed stimuli‐responsive DNA hydrogels incorporating aptamer‐functionalized Y‐DNA/L‐DNA networks and gold nanoparticle (AuNP) reporters.^[^ ^62^ ^]^ This system enabled visual thrombin detection through target‐induced hydrogel dissociation and AuNP release, demonstrating the potential of dynamic 3D sensing matrices.

Compared to traditional biosensors, DNA origami‐based biosensors offer broader target detection, longer lifespans, and lower production costs (**Table** **2**). Furthermore, DNA origami‐based biosensors possess several advantages: (I) DNA origami structures can be customized for specific scenarios and needs.^[^ ^57^, ^61^ ^]^ (II) DNA is easily synthesized chemically, allowing precise functionalization with fluorophores, redox reporters, or chemical linkers via automated oligonucleotide synthesis. However, challenges remain with DNA origami‐based biosensors: (I) Structural stability constraints: DNA nanostructures under physiological conditions (pH 5.8–7.4, 150 mM NaCl, 37 °C) are subject to random fluctuations, which may compromise biosensor performance by causing premature probe detachment from the origami scaffold, disrupting inter‐probe spatial coordination necessary for multiplex detection, and accelerating nuclease‐mediated degradation in biological matrices. (II) Biocompatibility limitations: Surface modifications for addressability and target‐specific recognition (e.g., aptamer functionalization) can trigger immunogenic responses, such as CpG motif‐induced TLR9 activation in antigen‐presenting cells, complement cascade initiation through interactions with the exogenous DNA backbone, and unintended binding with residual conjugation moieties (e.g., NHS esters, maleimide groups).

**Table 2:** Summary of applications and challenges of multifunctional DNA origami in biosensing.

	Applications	Advantages	Problems	Refs.
Biosensing	DNA origami as a probe recognition signal for the detection of specific DNA/RNA sequences	DNA origami‐based biosensors offer a wider range of detection targets, longer lifespans, and lower production costs. Biosensors with DNA origami structures can be tailored to meet specific needs and scenarios. The ease of modifying the DNA surface allows for the straightforward introduction of chemical linkers or fluorescent dyes at both ends, enabling versatile detection capabilities. The DNA origami structure serves as a signal amplification platform, significantly improving detection sensitivity. The DNA origami can realize highly specific recognition through base pairing, which is suitable for precise detection of target molecules.	DNA origami is prone to degradation in environments with high temperatures, strong acids and bases, or in the presence of nucleases, impacting the stability of the sensor. Complex samples in the environment may interfere with DNA origami sensor signals.	[58]
	Immobilization of antibodies or aptamers for detection of specific proteins or biomarkers by modification of DNA origami surfaces			[60]

Overcoming the three major barriers of stability, scale, and standardization is crucial for the clinical translation of DNA origami biosensors, driving their evolution from proof‐of‐concept demonstrations to practical applications. Currently, emerging solutions such as chemical modifications, structural crosslinking, artificial intelligence (AI)‐assisted design, and microfluidic assembly platforms address these challenges. Future research should prioritize enhancing the robustness of DNA origami biosensors and enabling their low‐cost manufacturing for deployment in complex biological environments, thereby facilitating widespread adoption. Additionally, exploring alternative programmable nanomaterials—including peptide nanostructures, RNA origami, and polymer scaffolds—can complement DNA origami's capabilities and accelerate functional advancements. Ultimately, the integration of synthetic biology, materials science and computational intelligence is the key to overcoming current limitations and unlocking the broad potential of DNA origami technology in biosensing and diagnosis.

### 3.5 Gene Editing Technology

The CRISPR/Cas9 system has become a cornerstone technology in synthetic biology, enabling precise genomic network engineering through programmable nucleases. Pioneering work by Sun et al. (2015) demonstrated the use of RCA‐synthesized DNA nanoclews for nucleus‐targeted delivery of sgRNA/Cas9 complexes, achieving site‐specific indels via Cas9 fused with a nuclear localization signal, without compromising cellular viability.^[^ ^63^ ^]^ This milestone showcased the feasibility of spatially controlled genome editing using synthetic DNA architectures.

**Figure 5:**
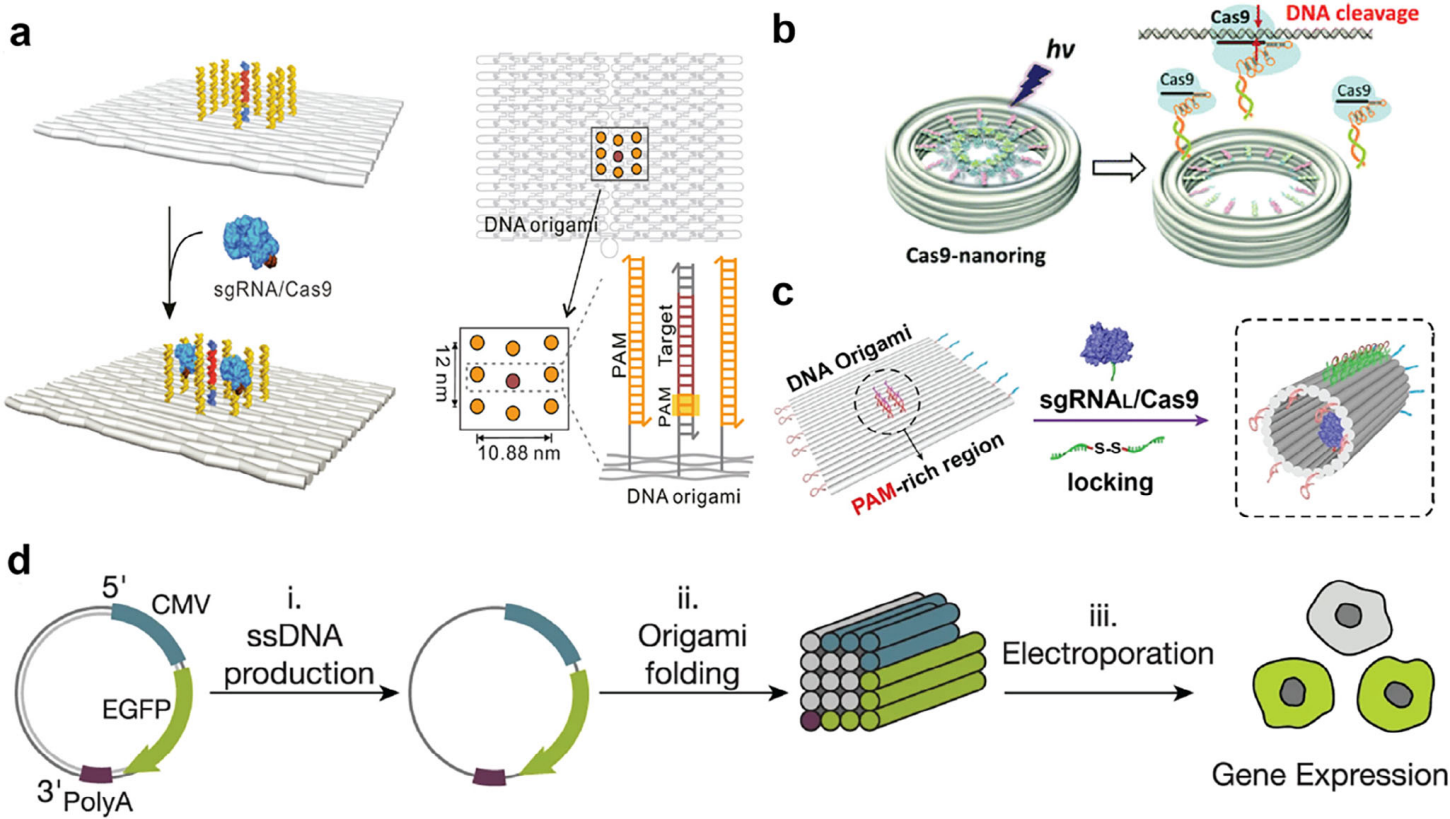
a) Schematic diagram of a PAM‐rich microenvironment constructed on DNA origami. Adapted with permission.^[^ ^64^ ^]^ Copyright 2020, The Authors, published by American Association for the Advancement of Science. b) The photo‐controlled sequence‐selective dsDNA introduces Cas9 into the cavity of the circular DNA origami and releases Cas9 through photoirradiation. Reproduced with permission.^[^ ^65^ ^]^ Copyright 1996–present, Royal Society of Chemistry. c) Efficient recruitment and loading of sgRNA/Cas9 complexes through precisely arranged PAM enrichment regions in DNA origami structures and pre‐designed DNA/RNA hybridization. Adapted with permission.^[^ ^66^ ^]^ Copyright 2023, Wiley‐VCH GmbH. d) Folding and expressing genes from DNA origami structures. Adapted under terms of the CC‐BY license.^[^ ^67^ ^]^ Copyright Year 2023, The Author(s), published by Springer Nature.

In recent years, as research on DNA origami and CRISPR systems has advanced, their integration has become more precise and efficient. The PAM (protospacer‐adjacent motif) site plays a critical role in CRISPR, as it recruits sgRNA/Cas9 to the target site and activates the nuclease domain of Cas9. Wang and colleagues employed DNA origami to program the microenvironment of the PAM site to study the DNA editing process.^[^ ^64^ ^]^ The “antennae effect” of PAM‐rich microenvironments may enhance gene editing efficiency with low concentrations of sgRNA/Cas9 (**Figure** **5**a). Investigating the effects of PAM antennae spatial distribution and density contributes to a deeper understanding of gene editing in complex environments, such as chromatin, and offers a new perspective on improving editing efficiency. Enhancing gene editing efficiency can also reduce delivery burdens and minimize impacts on cellular activity. Furthermore, effective packaging and delivery of the CRISPR/Cas9 system to tissues and cells is critical for successful gene editing. A promising technological solution to this challenge is the light‐controlled gene wiring technique, commonly used in synthetic biology. Katsuhiko Abe et al. utilized photo‐controlled sequence‐selective dsDNA to introduce Cas9 into the lumen of a ring‐shaped DNA origami, releasing Cas9 via photoirradiation (Figure 5b).^[^ ^65^ ^]^ Additionally, a “locking” structure can be incorporated into DNA origami to facilitate the targeted transport of the Cas9 system. For instance, Tang et al. demonstrated the efficient recruitment and loading of sgRNA/Cas9 complexes using DNA origami, with a precisely organized PAM‐enriched region and pre‐designed DNA/RNA hybridization. A locked strand with disulfide bonds (L‐DOPAMRC) was rolled up to achieve targeted transport and efficient endosome escape (Figure 5c).^[^ ^66^ ^]^ The escaped L‐DOPAMRC can then be opened by GSH reduction, with the sgRNA/Cas9 complex released through RNase H cleavage of the RNA strand in DNA/RNA hybrids.

CRISPR/Cas9‐based gene editing plays a pivotal role in gene therapy and holds significant promise in the field of synthetic biology (**Table** **3**). The genetic code embedded in DNA nanoparticles created through DNA origami or related technologies can be recognized and transcribed by RNA polymerase in vitro. Additionally, sculpted DNA nanoparticles can serve as substrates for Cas9‐mediated gene modification and gene expression in cell culture. Kretzmann et al., demonstrated that DNA origami folding can facilitate gene expression, under the condition that DNA origami can be unfolded in the intracellular environment. This is achieved through the targeted design of the helper backbone and the inclusion of elements such as the Kozak sequence, woodchuck post‐transcriptional regulatory element, and at least one inverted terminal repeat‐like (ITR) upstream of the expression cassette in a synthetic scaffold, enhancing gene expression efficiency (Figure 5d).^[^ ^67^ ^]^ Chang Yong Oh et al., explored the construction of self‐assembled DNA nanoparticles containing intact gene cassettes capable of expressing genes in vitro. They further studied how sculpted DNA nanoparticles could serve as substrates for Cas9‐mediated gene modification and gene expression in cell culture. This study focused on the impact of the structural domains of ssDNA and the configurations of genetically controlled elements (such as promoter and enhancer sequences) on gene expression in human cell lines.^[^ ^68^ ^]^ The research showed that the presence, relative density, and positioning of ssDNA structural domains significantly influence overall gene expression levels. Subtle variations in the folding structure of DNA nanoparticles can regulate the rate and/or level of gene expression. Tang et al. utilized circular single‐stranded DNA (Css DNA) as a conformationally switchable and highly efficient gene expression vector, with partial complementary DNA strands hybridizing to it to control the expression of regulatory genes. The addition of blocking strands inhibited the expression of genes from Css DNA transfected into mammalian cells via lipofection, and this inhibition was recovered by co‐transfecting triggering strands. This provides a theoretical basis for future exploration of other biologically relevant signals as triggers (such as RNAs, ions, pH, enzymes or other metabolites) for endogenous dynamic regulation of Css DNA as a gene regulator integrated into more complex and functional synthetic gene networks.^[^ ^80^ ^]^ Moreover, by incorporating fluorescent labeling or reporter gene systems, DNA origami structures can monitor gene expression levels in real‐time and dynamically adjust regulatory intensity through a closed‐loop design. In synthetic biology, such systems can be leveraged to construct smart gene circuits for the precise programming of cell behavior.

**Table 3:** Summary of applications and challenges of multifunctional DNA origami in gene editing system.

	Applications	Advantages	Problems	Refs.
Gene editing system	DNA origami programming of PAM sites	DNA origami, with its molecular and submolecular precision and addressability, offers significant advantages in studying biomolecular interactions at the single‐molecule level. It enables simple detection using optical and atomic force microscopy (AFM).	Efficient and low‐cost preparation strategies are essential for the development of delivery systems based on nucleic acid nanostructures. Since DNA is a substrate for various nucleic acid hydrolases inside and outside the cell, biostability needs to be improved. High doses of nucleic acid nanostructures may induce potentially immunogenic stimuli.	[18]
	DNA origami wraps Cas9 protein for efficient transport	Efficient targeted transport of Cas9 systems is enabled by attaching a “locking” structure to DNA origami. Safe, specific, and non‐pathogenic.		[68]
	DNA origami nanoparticles are expressed in the intracellular environment and regulate the rate and/or level of gene expression	DNA origami nanoparticles offer unique advantages in precision control, programmability, and versatility within cellular systems. These capabilities enable the construction of smart gene circuits for the precise programming of cellular behavior.		[19]

DNA origami, with its programmable nanoscale spatial positioning capability, offers a unique and precise platform for gene editing systems. By precisely programming PAM antennas on the DNA origami platform and folding special sequences in DNA origami, the efficiency of gene editing can be enhanced, and even the influence of various design parameters of DNA origami nanoparticles on gene expression in mammalian cell culture is gradually being studied. These studies confirm DNA origami's potential as a versatile platform technology in biomedicine, synthetic biology, and related fields. However, broader application faces significant challenges, including insufficient structural stability within living cells, immunogenicity, and the high cost of large‐scale production. Notably, structural properties such as mechanical strength impact immunogenicity, suggesting that more mechanically robust DNA origami structures may also exhibit greater resistance to nucleases.^[^ ^85^ ^]^ Encapsulation strategies (e.g., lipid nanoparticles, hydrogels) are being explored to enhance in vivo stability and enable specific targeting. Furthermore, integrating computational learning allows for balancing multiple parameters to design inherently more stable DNA origami. Future research should prioritize optimizing self‐assembly processes (e.g., developing dynamically responsive origami structures), integrating multimodal systems (combining activation mechanisms like optogenetic control, enzyme drives, and beyond), and achieving breakthroughs in in vivo in situ assembly. Overcoming these challenges is crucial for advancing intelligent, diagnostics‐therapeutics‐integrated nanomachines based on DNA origami technology.

### 3.6 Cell‐Free Synthetic Biology

To streamline the design process and facilitate the debugging of complex synthetic circuits, cell‐free synthetic biology has gained widespread adoption across both academia and industry.^[^ ^69^ ^]^ This approach offers a novel means of understanding biological systems, enabling the synthesis of new biological systems beyond natural biological constraints by utilizing and controlling biomass.

Leveraging the predictable self‐assembly properties of DNA, it has become a key tool in building multifunctional molecular machines and structures. Notably, the advancement of structural and dynamic DNA nanotechnology has significantly impacted cell‐free synthetic biology for synthetic cells.^[^ ^70^ ^]^ In this field, DNA structures with precise shapes and sizes can act as miniature reaction vessels, partitioning metabolic processes. Faithful mimicry of biochemical systems requires the introduction of metabolic compartments. By designing DNA origami structures with specific geometries, various reaction components (e.g., DNA templates, RNA polymerase, ribosomes) can be confined to defined spaces, akin to how organelles create independent environments for specific biochemical reactions. Huang et al. designed two interconnected DNA origami nano‐compartments (measuring 25 nm × 41 nm × 53 nm each) for regulated protein unfolding and degradation.^[^ ^71^ ^]^ These spatial constraints accelerated individual reaction rates by a factor of tenfold, while the physical connection of the split enzyme to the chimera efficiently coupled the two reactions and minimized off‐target interactions. This also demonstrates the potential utility of methods for engineering biocatalytic pathways on selected substrates. A key advantage of compartmentalization is that metabolic reactions can be isolated within specific compartments, with concentration gradients between them providing further opportunities for optimization. The ability of DNA origami structures to integrate multiple functional elements simultaneously offers significant advantages for compartmentalized applications. Biomolecular motors in living cells, such as myosin, kinesin, and kinetochore proteins, move along specific cytoskeletal filaments to assemble, sort, or localize at designated sites within the cell. Dynamic molecular machine DNA walker‐origami systems can be used to simulate key processes such as material transport, signal transduction or metabolic pathway coordination within cells. The selective surface functionalization capability of the DNA origami substrate further confers significant potential for functional versatility. Within this system, the DNA walker transports molecular cargo along designated paths via anchor strand displacement. Directional translocation of the walker is driven by diverse mechanisms, such as strand displacement reactions, enzymatic cleavage (mediated by proteases or DNases), photoactivation, and chemical stimulation.^[^ ^86^, ^87^ ^]^ Liu et al. demonstrated the successful integration of a repeatable, light‐driven, non‐bridge‐burning DNA molecular motor within a DNA origami scaffold, achieving autonomous directional motion.^[^ ^84^ ^]^ This integrated system synergistically exploits the dynamic positioning capability of molecular motors at the nanoscale and the structural programmability inherent to DNA origami, holding significant potential for enabling diverse dynamic functions within biological systems.

DNA origami technology introduces an innovative approach for enzyme immobilization, enhancing enzyme stability. In cell‐free systems, free enzymes are susceptible to inactivation due to environmental factors such as pH fluctuations and temperature changes.^[^ ^72^ ^]^ However, when enzymes are immobilized on DNA origami structures, the DNA scaffold provides protective support, allowing the enzyme to maintain activity under relatively harsh conditions.^[^ ^73^ ^]^ The enzyme activity can be further modulated by altering the conformation of the DNA origami structure. For instance, DNA origami can be designed to respond to specific environmental signals, such as changes in ion concentration or light exposure.^[^ ^74^ ^]^ Upon such environmental changes, the DNA origami structure undergoes conformational shifts, influencing the accessibility of the enzyme's active site and enabling precise regulation of its activity. This form of regulation is crucial for controlling complex metabolic pathways in cell‐free synthetic biology.

In cell‐free synthetic biology, assembling multiple biomolecules (e.g., proteins, nucleic acids) in specific orientations is essential for forming functional complexes. DNA origami structures serve as precise templates, guiding the assembly of these biomolecules. For example, in constructing artificial ribosomes, DNA origami can direct the assembly of ribosomal subunits, associated protein factors, and RNA molecules according to the structural patterns of natural ribosomes, thereby enhancing the efficiency and functional activity of the artificial ribosome. Additionally, DNA nanoparticles can be easily transcribed by T7 RNA polymerase in vitro.^[^ ^75^ ^]^ These findings highlight the significant potential of sculpted, gene‐carrying, self‐assembled DNA nanoparticles in biomedical and related applications. The integration of T7 RNA polymerase and DNA templates on DNA origami platforms enables the construction of highly efficient and spatially confined genetic reaction systems, allowing precise control over the coordination and efficiency of biological reactions.^[^ ^81^, ^82^ ^]^This advancement establishes a critical technical foundation for the design and development of large‐scale, programmable artificial synthetic biological systems, such as high‐performance biomolecular factories and synthetic genetic circuits.

One of the primary goals of cell‐free synthetic biology is to create complete synthetic cells that can efficiently and cost‐effectively produce target molecules, while minimizing metabolic burdens, avoiding unwanted metabolic pathways, and overcoming organism‐specific limitations. The main challenge in cell‐free synthetic biology remains the integration of biomolecular mechanisms into larger, more complex systems. Achieving this integration is essential to understanding how molecules interact when combined, identifying cooperative links between molecular mechanisms, and assessing the robustness of biochemical systems. The intersection of DNA origami and cell‐free synthetic biology presents a promising area that not only offers the potential to unravel the mysteries of life but also holds significant promise in advancing metabolic production (**Table** **4**).

**Table 4:** Summary of applications and challenges of multifunctional DNA origami in gene editing cell‐free synthetic biology.

	Applications	Advantages	Problems	Refs.
cell‐free synthetic biology	DNA origami used to build versatile molecular machines and structures	Precise and predictable self‐assembly properties of DNA for greater flexibility in building versatile molecular machines.	The core challenges of DNA origami to build multifunctional molecular machines lie in functional synergy, energy efficiency and bio‐adaptability. Immobilization of biomolecules on DNA origami may disrupt their active conformation (e.g., enzyme active sites are obscured).	[71]
	DNA origami guides self‐assembly of biomolecules	DNA origami structures can provide precise templates to guide the assembly of these biomolecules.		
	Introducing metabolic compartments by designing shape‐specific DNA origami structures	Providing relatively independent reaction environments similar to organelles for specific biochemical reactions. Compartment surfaces can be modified with targeting molecules, environmental response elements or signaling receptors to achieve “compartment‐sensor‐effector” integration. DNA itself is a natural biomolecule. The degradation products are not harmful to the cell and are more easily metabolized and removed.	DNA origami delivery efficiency is low, and the breakthrough direction lies in the use of intracellular DNA replication and repair enzymes for compartmentalized in situ synthesis. DNA origami compartments cannot be equally distributed with cell division, leading to functional compartmentalization failure. Natural organelles can be dynamically renewed, while DNA structures cannot be repaired autonomously after damage. CRISPR‐based DNA repair systems can be developed in the future to repair DNA origami structures in situ.	[72]
cell‐free synthetic biology	DNA origami immobilizes enzyme to improve enzyme stability	Sequence‐programmable DNA architectures enable precise localization and spatial requirements of enzymes for improved catalytic optimization. DNA origami has high mechanical stability and effectively protects enzyme molecules. The surface of DNA origami can be easily modified to introduce functional groups or targeting molecules, enhancing enzyme targeting and catalytic efficiency. The DNA origami structure provides multiple binding sites, enabling the simultaneous immobilization of multiple enzyme molecules.	The immobilization process may affect the natural conformation of the enzyme, diminishing activity. Real‐time monitoring of catalytic processes of immobilized enzymes still faces technical challenges.	[73]
	DNA origami can be transcribed in vitro by T7 RNA polymerase	The great plasticity of structure and chemistry of self‐assembled DNA nanoparticles.	Multiple challenges persist, such as structural stability, transcription efficiency, and sequence design.	[75]

## 4 Conclusion and Outlook

The bottom‐up development of increasingly complex synthetic cells has enhanced our understanding of natural cells. Alongside rapid advancements in form and function simulation, artificial cells offer numerous potential applications in synthetic biology. As one of the DNA nanotechnologies, DNA origami has emerged as a crucial supportive technology for synthetic biology, showing great promise in the construction of artificial synthetic cells.

This review focuses on exploring the application potential of DNA origami in synthetic biology from two dimensions: extracellular to intracellular and cell‐free synthetic biology. DNA origami technology enables the construction of spatially addressable nanostructures through programmed self‐assembly of nucleic acids. At biological interfaces, this technique can reshape lipid membranes via frame‐guided assembly, modify the biomimetic cell membrane function of liposomes, or reconstitute membrane channels for regulated transport in response to environmental changes. Furthermore, DNA origami objects can be assembled into higher‐order architectures featuring larger pores or multiple parallel channels, facilitating detailed observation of substance transport processes. DNA origami fixed on the cell membrane can mediate intercellular communication by carrying signal molecules (such as aptamers and antibodies) or chain shift reactions, simulating natural signal transduction pathways. In emulating the cytoskeleton, the rigid DNA framework provides structural support to cells and guides the spatial organization of intracellular proteins. Its protein network assembly ability can precisely organize multi‐enzyme cascade reaction pathways, significantly enhancing metabolic efficiency. As highly sensitive biosensors, DNA origami structures incorporate probe molecules to achieve precise signal detection. DNA origami offers programmable PAM sites for enhanced editing efficiency and enables spatiotemporally controllable gene editing through sophisticated “lock” mechanisms. Such capabilities underpin the development of intelligent gene circuits for precise programming of cellular behavior. In cell‐free synthetic biology, DNA origami skeletons provide standardized scaffolds for multifunctional molecular machines and structures, efficiently organize cell‐free expression systems, and promote the development of synthetic biological circuits and molecular factories. These cross‐scale applications demonstrate the powerful potential of DNA origami as a “molecular canvas” in integrating life system modules and reshaping biological functions.

Despite its potential, there are several challenges in applying DNA origami technology in synthetic biology, mainly focusing on stability, large‐scale production and design complexity: (I) The sensitivity of DNA origami nanostructures to environmental factors (e.g., pH, ionic strength, temperature, nucleases like DNase I) can affect their performance in different environments, compromising their durability and functionality.^[^ ^76^, ^77^, ^78^ ^]^ These studies elucidate degradation mechanisms while strategies rapidly diversify. Both chemical modifications/protective coatings and structural cross‐linking can enhance the resistance of DNA origami structures to nuclease degradation. L‐DNA nanostructures (unnatural left‐handed chirality) exhibit superior nuclease resistance and biological stability over conventional D‐DNA (conventional right‐handed DNA).^[^ ^88^ ^]^ Furthermore, structural stability within DNA origami under physiological conditions can be enhanced through cross‐linking strategies, such as inducing site‐specific covalent cyclobutane pyrimidine dimer (CPD) bond formation or click chemistry‐mediated covalent closure via ultraviolet irradiation.^[^ ^89^, ^90^ ^]^ Additionally, various protective coatings, including virus‐mimicking lipid bilayers, and cationic polymer modifications (e.g., polyethylene glycol (PEG)‐oligomers, chitosan, polyethyleneimine (PEI)), significantly bolster the resistance of DNA origami to destabilization under low‐salt conditions, degradation by DNase I, and serum‐mediated digestion.^[^ ^91^, ^92^, ^93^ ^]^ (II) While DNA origami synthesis is feasible at the laboratory scale, large‐scale production remains a challenge, limiting its widespread application in industrial and medical fields. Utilizing engineered cellular factories, such as amplified phage or modified bacterial systems, for the production of large‐scale ssDNA scaffolds (e.g., M13mp18) or custom fragments represents a highly promising route.^[^ ^94^ ^]^ The core challenge lies in the optimization and quality control of scaled‐up bioprocesses. Additionally, the application of microfluidic chip technology for high‐throughput, scalable mixing and assembly control shows significant potential to increase throughput, reduce costs, and improve consistency. Using microfluidic double‐emulsion droplets as nano‐reactors with osmotically controlled DNA concentration, Chen et al. achieved highly efficient (up to 98.6% success), size‐controllable (19.3–56.8 µm) assembly of single DNA nanostructures per droplet.^[^ ^95^ ^]^ (III) While pursuing higher spatial precision, richer functional integration (e.g., logic gates, actuators), and more complex dynamic behaviors, ensuring design success rates, predictability, and ultimate production efficiency remains an ever‐growing challenge. Fundamental design translates shapes into scaffold/staple sequences, aided by progressively automated software: from manual routing (caDNAno) to automated sequence generation (DAEDALUS) to multifunctional platforms (Adenita) enhancing versatility.^[^ ^13^, ^96^, ^97^, ^98^ ^]^ Innovatively, Wang et al. developed BRAIDS, an automated design process that eliminates traditional scaffold dependence in DNA nanostructures. Through localized self‐assembly of short strands, BRAIDS allows arbitrary construction of large‐scale, highly complex 2D/3D DNA wireframe nanostructures.^[^ ^99^ ^]^ Based on such technologies, a standardized ‘DNA origami component library’‐containing functionalized vertices, edges, scaffolds, dynamic elements, and capping sequences‐alongside associated design rules will facilitate modular assembly, enabling system‐level engineering and field standardization. This approach embodies synthetic biology principles (standardization, modularity, predictability) for building complex systems. Ouldridge et al. developed the coarse‐grained OxDNA model, capturing DNA's mechanical/thermodynamic properties for nanostructure simulation.^[^ ^100^ ^]^ Snodin et al. later expanded it to oxDNA2, enabling quantitative prediction of structural mechanics (persistent length, helicity) and salt‐ion sensitivity via a refined parameter library.^[^ ^101^ ^]^ A standardized modular library embodies core synthetic biology principles‐standardization, modularity, abstraction, and predictability‐to build complex systems. This significantly boosts DNA nanotechnology's design efficiency and productivity while accelerating its applications in precision medicine, molecular computing, and smart materials. Ultimately, it enables “programming” the material world, akin to synthetic biology's engineering of biological systems.

Single‐molecule folding appears more promising, with significant advances in ssDNA origami and RNA origami. RNA nanostructures offer unique advantages over DNA: functional diversity, cost‐efficient gene expression, and intracellular adaptability.^[^ ^102^ ^]^ Furthermore, as molecules with biological homology, Self‐assembled peptide nanomaterials exhibit high biocompatibility, diverse functionalities, and biomedicine potential, though limited by physiological instability.^[^ ^103^, ^104^ ^]^ Based on their self‐assembly capabilities, peptide nanomaterials offer immense potential applications in biomedicine, biotechnology, and drug delivery. Other polymeric scaffolds such as dendrimers, liposomes, and protein cages exhibit significant advantages in drug loading capacity, scalability for mass production, cost, and potential in vivo delivery efficiency (e.g., lipid nanoparticles). However, they fall far short of DNA origami in achieving atomically precise spatial organization and complex programmable behavior.^[^ ^105^ ^]^ DNA origami's core strengths remain unparalleled spatial precision, predictable assembly, and structural programmability, despite challenges. Future breakthroughs may derive from hybrid systems integrating these materials for functional complementarity.

Deep integration of DNA origami with emerging technologies accelerates its maturation. Exploring novel design spaces using artificial intelligence (AI) represents a key future focus. AI enables automated sequence design, multi‐objective optimization, and prediction of folding thermodynamics, facilitating robust nanostructures with reduced trial‐and‐error costs. Leveraging AlphaFold 3's AI structure prediction capabilities enables the efficient design and optimization of complex DNA nanostructures, dramatically reducing the traditional reliance on costly trial‐and‐error methods. This advancement has shifted the paradigm for DNA nanounit design from empirical approaches to AI‐predictive modeling, establishing an efficient and scalable new paradigm for programmable biomolecular self‐assembly.^[^ ^106^ ^]^ Do‐Nyun Kim et al. employed graph neural networks (GNNs) to overcome training data scarcity for 3D structure prediction.^[^ ^107^ ^]^ Integrating high‐throughput screening platforms with DNA origami technology enables parallel analysis and evaluation of vast numbers (tens of thousands to millions) of assembly reaction conditions or samples within short timeframes, rapidly identifying optimal combinations and structurally sound products.^[^ ^108^ ^]^ Microfluidics permits “on‐chip” production‐from fabrication to purification—advancing scalable manufacturing.^[^ ^109^, ^110^ ^]^ Such integration establishes DNA origami as a precision engine for applications like programmable endothelial barriers that control integrin‐ligand interactions in cancer drug screening.^[^ ^111^ ^]^

Even if technical challenges are resolved, current DNA origami still faces translational hurdles in practical applications‐particularly in in vivo medical settings. The first hurdle is immunogenicity. Lucas et al. demonstrated the biosafety of DNA origami under high‐dose repeated administration, showing only predictable mild immunogenicity and no significant in vivo toxicity.^[^ ^85^ ^]^ This supports its safety and potential as a biomedical platform, particularly for drug delivery and vaccines. Moreover, studies indicate that DNA origami can precisely control the nanoscale spatial arrangement of immunostimulants (e.g., CpG oligonucleotides), significantly boosting cancer vaccine immune responses and antitumor immunity.^[^ ^112^ ^]^ However, the immune responses triggered by DNA origami in vivo remain poorly understood. Deepening this understanding, particularly its immunogenicity, is crucial for clinical translation. Furthermore, critical pharmacokinetic aspects like in vivo distribution, circulation half‐life, hepatosplenic/renal clearance, and tumor targeting efficiency (EPR effect‐dependent) warrant deeper investigation. As a complex platform for nanomedicine and diagnostics, DNA origami requires comprehensive safety evaluation (toxicity profiles, genotoxicity, immunotoxicity) and scalable GMP manufacturing to meet regulatory standards (FDA, EMA). Establishing rigorous material characterization, analytical methods, and design‐function‐behavior relationships is essential for a robust regulatory framework. Overcoming these technical and translational challenges will enable the practical use of DNA origami in synthetic biology and precision nanomedicine.

## Conflict of Interest

The authors declare no conflict of interest.
